# Correction: Soares, D.J. Bridging a Century-Old Problem: The Pathophysiology and Molecular Mechanisms of HA Filler-Induced Vascular Occlusion (FIVO)—Implications for Therapeutic Interventions. *Molecules* 2022, *27*, 5398

**DOI:** 10.3390/molecules29246006

**Published:** 2024-12-20

**Authors:** Danny J. Soares

**Affiliations:** 1 American Foundation for Aesthetic Medicine (AFFAM), Fruitland Park, FL 34731, USA; drsoares@plasticsurgeryvip.com; 2 College of Medicine, University of Central Florida, Orlando, FL 32827, USA

## Figures Sources Information Missing 

In the original publication [1], the references for Figures 8 and 18 were missing. The updated Figures 8 and 18 captions should read as follows:

**Figure 8.** The two types of histomorphology of the facial superficial musculoapo-neurotic system (SMAS), according to Ghassemi et al. [127]: (**a**) Type I morphology is found predominantly in the cheek, forehead, and lateral face and features a uniform layer of adipose tissue interspersed amidst the fibrous septa, spanning the space between the SMAS and the dermis. (**b**) Type II morphology is present in the central facial tissues of the perioral and perinasal regions and is characterized by thick fibromuscular inser-tions emanating from the SMAS directly into the dermis, forming a more rigid adhesion between the two planes. Adapted from the classification by Ghassemi et al., *Aesthetic Plastic Surgery*, published by Springer Nature, 2003 [127], with permission.

**Figure 18.** The clinical stages and appearance of the skin following an ischemic in-jury caused by vascular occlusion with dermal fillers, according to Murray et al. [86]: Stage I occurs immediately and is characterized by skin blanching with delayed capillary refill. Stage II manifests over 72 h, with livedoid skin changes due to dermal pooling of deoxygenated blood in underperfused regions. Stage III is characterized by the func-tional deterioration of the skin barrier with partial desquamation and overgrowth of cutaneous flora. Stage IV occurs 5–10 days post injury and is characterized by coagulative necrosis, with the eventual formation of an eschar (Stage V) which may persist for weeks. Adapted from the classification by Murray et al., *The Journal of Clinical and Aesthetic Dermatology*, published by Matrix Medical Communications, 2021 [86], with permission.

The authors state that the scientific conclusions are unaffected. This correction was approved by the Academic Editor. The original publication has also been updated.
