# Supplementary material for: Bridging a Century-Old Problem: The Pathophysiology and Molecular Mechanisms of HA Filler-Induced Vascular Occlusion (FIVO)—Implications for Therapeutic Interventions
Source: Molecules. 2022 Aug 24;27(17):5398. doi: 10.3390/molecules27175398 (PMC9458226; doi:10.3390/molecules27175398)
Supplement: Supplementary file 1 [file molecules-27-05398-s001.zip › Video Legends.pdf]

Videos Legends:

Video 1. Type I dissemination of polymethylmethacrylate filler within the central auricular artery in the rabbit ear model. The filler completely disperses distally as it is introduced into the artery. Adapted from Nie et al. [151].

Video 2. Type IV dissemination of hyaluronic acid filler within the central auricular artery in the rabbit ear model. The filler forms an elongating intraluminal plug that completely occludes the artery and partially disperses distally. The filler plug additionally extends proximally to eventually enter and occlude upstream branches as well (arrowhead). Adapted from Nie et al. [151].

Video 3. Dynamic state of an intraluminal plug of polymethylmethacrylate filler bolus shortly following inoculation into the central auricular artery in the rabbit ear model. The filler is gradually deformed, fragmented, and dispersed before settling into an equilibrium. Adapted from Nie et al. [151].
